# Automated highway pavement crack recognition under complex environment

**DOI:** 10.1016/j.heliyon.2024.e26142

**Published:** 2024-02-15

**Authors:** Zhihua Zhang, Kun Yan, Xinxiu Zhang, Xing Rong, Dongdong Feng, Shuwen Yang

**Affiliations:** a Faculty of Geomatics, Lanzhou Jiaotong University, Lanzhou, China; b National-Local Joint Engineering Research Center of Technologies and Applications for National Geographic State Monitoring, Lanzhou, China; c Gansu Provincial Engineering Laboratory for National Geographic State Monitoring, Lanzhou, China; d Gansu Province Key Laboratory of Highway Network Monitoring, Lanzhou, China

**Keywords:** Highway pavement crack, Residual network, Convolutional block attention module, Recognition

## Abstract

The pavement is vulnerable to damage from natural disasters, accidents and other human factors, resulting in the formation of cracks. Periodic pavement monitoring can facilitate prompt detection and repair the pavement diseases, thereby minimizing casualties and property losses. Due to the presence of numerous interferences, recognizing highway pavement cracks in complex environments poses a significant challenge. Nevertheless, several computer vision approaches have demonstrated notable success in tackling this issue. We have employed a novel approach for crack recognition utilizing the ResNet34 model with a convolutional block attention module (CBAM), which not only saves parameters and computing power but also ensures seamless integration of the module as a plug-in. Initially, ResNet18, ResNet34, and ResNet50 models were trained by employing transfer learning techniques, with the ResNet34 network being selected as a fundamental model. Subsequently, CBAM was integrated into ResBlock and further training was conducted. Finally, we calculated the precision, average recall on the test set, and the recall of each class. The results demonstrate that by integrating CBAM into the ResNet34 network, the model exhibited improved test accuracy and average recall compared to its previous state. Moreover, our proposed model outperformed all other models in terms of performance. The recall rates for transverse crack, longitudinal crack, map crack, repairing, and pavement marking were 88.8%, 86.8%, 88.5%, 98.3%, and 99.9%, respectively. Our model achieves the highest precision of 92.9% and the highest average recall of 92.5%. However, the effectiveness in detecting mesh cracks was found to be unsatisfactory, despite their significant prevalence. In summary, the proposed model exhibits great potential for crack identification and serves as a crucial foundation for highway maintenance.

## 1 Introduction

The presence of pavement cracks on highways can have detrimental effects on driving comfort and traffic safety. Therefore, the rapid and accurate detection of the pavement cracks is a significant measure for ensuring road safety. Presently, highway maintenance organizations employ detection trucks to capture high-resolution images of pavement fractures prior to implementing costly, time-consuming, and labour-intensive artificial marking techniques. Image processing technology has demonstrated a certain degree of potential in enhancing the efficiency of pavement crack identification [1,2]. Various threshold-based approaches have been employed for detecting the crack region with notable success [[3], [4], [5], [6]]. By employing various thresholds, cracks can be readily identified as they exhibit higher light absorption compared to other areas, resulting in darker spots within the image [7]. Nevertheless, these methods frequently demonstrate sensitivity to noise and heavily rely on the selection of appropriate thresholds. The detectability of fractured pixels is significantly reduced by darker pixels in the presence of image noise. Some researchers have successfully identified cracks employing feature descriptors. For instance, Gabor filters [8,9] and wavelet transform [10] have been used to detect simple cracks. However, their performance is worse for alligator cracks, block cracks, and other complex shaped cracks and noisy within images.

By effectively reducing image noise and strengthening the contrast between the crack and the backdrop, other researchers have achieved remarkable success in crack detection [[11], [12], [13], [14]]. Nevertheless, their perspective on the crack remains limited as they solely extract the crack information from individual pixels, machine learning algorithms have also been used to detect cracks. The aforementioned issues are effectively resolved by employing the crack recognition method based on the random forest algorithm, thereby considerably enhancing the accuracy of recognition [15]. However, at low-level feature maps, it lacks the capacity to effectively discriminate between the crack and the background. While it can proficiently eliminate noise and learn the internal structure of cracks through manual selection of crack features, this method fails to consider diverse types of crack extraction. By conducting a comprehensive literature review, we systematically compare the merits and drawbacks of edge detection techniques, filter morphology approaches, and machine learning methods in the context of crack image processing, as illustrated in Table 1.

**Table 1:** Advantages and disadvantages of crack image processing methods.

method	advantage	disadvantage
Edge detection [14,16]	Highlight the edge structure in the image. Reduce computational complexity.	Sensitive to noise. Cannot recognize the meaning of cracks inside & outside the edge line. Easily disturbed by environmental factors.
Filter morphology [14,17,18]	Simple and easy to implement. Remove small details and noise from the image.	Resulting in excessive smoothing of the image. Not enough to handle complex textures in images.
Machine learning [14,19,20]	Automatic feature learning. Good adaptability to complex crack structures.	Requires many training samples. High computing resource requirements.

Based on the aforementioned issues, we conducted experiments and analyses to evaluate the performance of various models including VGG16, GoogleNet, MobileNet, ResNet18, ResNet34, Resnet50, ResNet34_AC and ResNet34_SE in crack recognition [[21], [22], [23], [24]]. Ultimately, we selected ResNet34 for improvement by incorporating CBAM and optimizing the loss function. The enhanced ResNet34-CBAM exhibited commendable accuracy and recall rates.

## 2 Related works

Numerous techniques have been proposed by Researchers for crack recognition, leveraging the advancements in deep learning technology such as deep convolutional neural networks (DCNNs) [[25], [26], [27]]). The application of convolutional neural networks (CNNs) can be traced back to the 1990s research conducted by Lecun et al. in the field of handwritten digit recognition [28]. He proposed the LeNet architecture, which served as a prototype of CNN. AlexNet [29] proposed by Alex et al. and VGGNet [30] proposed by Simonyan et al., which employed the stacked deepening convolutional layer approach, significantly advanced the progress of CNNs. The problem of vanishing gradients was addressed by He et al. [31] in 2015 through the proposal of a residual network, which effectively deepens the network and improves classification performance. Dorafshan et al. [32] constructed the SDNET2018 crack image dataset, which is designed to facilitate engineers and technicians in their application, and employed CNNs for accurate identification of cracks.

The hybrid identification method, integrating CNNs and edge detection, achieves superior performance compared to conventional edge detection methods [33]. Ni et al. [34] created a crack contour network (CDN) based on the CNN framework, which employed convolution feature fusion and pixel-level classification to automatically process apparent cracks in structures. However, it should be noted that the datasets used for evaluation primarily consisted of concrete pavement backgrounds. As a result, the approach exhibits inferior performance in terms of crack recognition when applied to asphalt road surfaces. Tong et al. [35] used a CNN for automated detection of cracks in asphalt roads. While the findings show that the CNN has superior stability and recognition accuracy compared to traditional detection algorithms (94.36%), albeit limited to identifying longitudinal cracks exclusively. Consequently, this approach is not readily applicable to alligator cracks, block cracks, and other cracks characterized by intricate geometries. The CrackNet model, proposed by Zhang et al. [36], aims to automate the detection of pavement cracks in images of asphalt surfaces. This model enables pixel-level crack detection, however, it does not provide subcategories for different types of cracks, making successful identification of hairline cracks challenging.

Vincenzo Dentamaro et al. [37] proposed AUCO ResNet, a Covid-19 pre-screening network designed for cough and respiration analysis. This architecture incorporates a trainable Mel-like spectrogram layer to fine-tune the Mel-like-Spectrogram, enabling the capture of relevant time-frequency information. Additionally, this technique is employed to weight local and global feature descriptors that specifically focus on high-frequency details. Shahzeb Khan Gul et al. [38] proposed a novel learning-based approach that utilizes an attention mechanism (AM) to synthesize novel views of a light-field image using a sparse set of input views (i.e., four corner views) captured by a camera array. To achieve final adaptive image refinement, they employed a residual convolutional block attention module (CBAM). Sequentially applied in the channel and spatial dimensions, attention modules facilitate learning and emphasize important features within the image.

The CNN-based crack profile network can automatically handle structural cracks by fusing convolutional features and performing pixel-level classification [39]. The random forest algorithm has significantly improved the accuracy of crack recognition, but it lacks the ability to distinguish between cracks and background in low-level feature maps [40]. Although machine learning methods can effectively eliminate noise and learn the internal structure of cracks by manually selecting crack features, they fail to consider different types of crack feature extraction [13]. Most supervised and semi-supervised machine learning models are considered as black box models due to their encapsulation of internal learning methods [[41], [42], [43]]. Despite the ability to detect pavement cracks at the pixel level, detecting small cracks remains challenging due to a lack of comprehensive techniques.

The progress achieved by CNNs in crack identification remains delicate when it comes to recognizing cracks in complicated environments. Factors such as pavement backdrops with marking paint, repairs, and varying degrees of wear and tear can directly impact the precision of recognition. Additionally, the presence of interference between transverse cracks, longitudinal fractures, and map cracks further exacerbates the difficulty of identification. To address these challenges, this paper employs transfer learning to train the ResNet34 model and integrate a CBAM into it. Then we conducted a comparative evaluation of our proposed model against state-of-the-art models including VGG16, GoogleNet [44], MobileNet [45], ResNet18, ResNet34, and ResNet50 models to evaluate its performance.

## 3 Methods

### 3.1 ResNet34

#### 3.1.1 ResNet structure

ResNet, a residual sub-network proposed by He-kaiming et al. [31], enables the construction of deeper networks through the stacking of these sub-networks. This network achieved the highest accolade in the ImageNet competition for its exceptional performance in classification, target detection, and picture segmentation tasks. Additionally, it secured first place for target detection and picture segmentation on the COCO dataset that year. The experiment shows that the residual network exhibits a higher degree of optimization simplicity, and increasing its depth can enhance accuracy while effectively mitigating the deterioration problem associated with introducing additional layers. Consequently, this leads to an improvement in overall network performance. However, it is worth noting that as the number of network layers increases, there tends to be a saturation or even degradation in training accuracy, which represents a significant challenge.

**Fig. 1:**
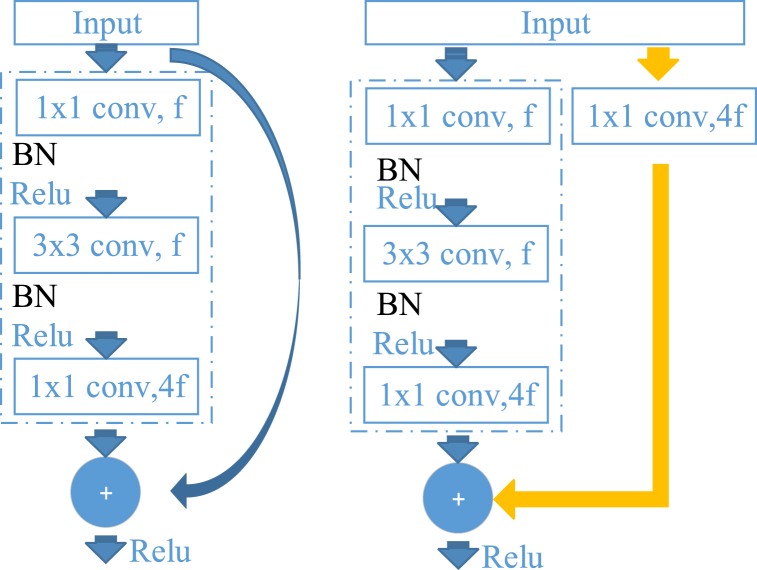
ResNet block. ResNet primarily incorporates direct channels into the network architecture, facilitating the seamless transmission of unprocessed input information to subsequent layers while preserving a proportion of the output from the preceding layer.

The central concept of ResNet is the residual module, as illustrated in Fig. 1. Each residual module consists of two pathways, one represents the identity mapping directly connected to the input features, while the other pathway convolves the input features to acquire the residue of feature map. The features from both pathways are aggregated through summation, resulting in the fundamental structure of ResNet comprises a 1 × 1 convolutional layer, a 3 × 3 convolutional layer, another 1 × 1 convolutional layer, and a shortcut connection layer. This structure is iteratively replicated until the emergence of a classifier, namely the average pooling layer (refer to Fig. 1). The activation function of the second 1 × 1 convolutional layer is linear, while the remaining layers employ nonlinear activation functions. This specific design choice ensures that the output feature dimensions match those of the input feature, enabling addition of features from two paths prior to applying the ReLU activation function, as depicted in Fig. 1 [46].

During the training of deep neural network models, the increase in the number of network layers often lead to gradient dispersion and gradient explosion, impeding network convergence and resulting in the degradation of model network performance instead of an improvement in accuracy. ResNet networks offer enhanced capabilities in addressing this problem by incorporating the residual module into the CNN and constructing deep network models through identity mapping, ensuring that the training error of the deep network does not exceed that of the shallow ones, Consequently, ResNet significantly increases the depth of deep neural networks.

**Fig. 2:**
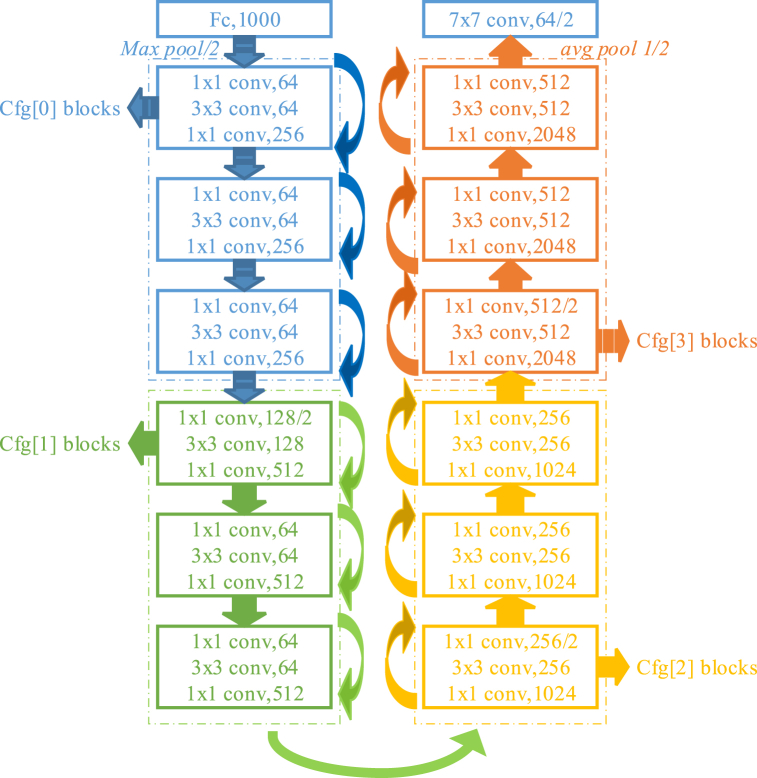
The network structure of ResNet; distinct colors denote residual modules with varying channel configurations.

The ResNet network structure is shown in Fig. 2, where different colors denote residual modules with varying channel configurations. In our experiment, we employed ResNet18, ResNet34, and ResNet50 networks. These three networks share the same structure but the differ in terms of the number of residual modules.

#### 3.1.2 ResNet34 structure

In the aforementioned ResNet structure, 18, 34, and 50 refer to the network structure with varying depths. Here, the depth refers to the number of layers that require parameter updates during training, such as convolutional layer and fully connected layer. Notably, ResNet18 and ResNet34 are considered shallow networks constructed based on BasicBlock. ResNet50 is a deep neural network structure that is established based on Bottleneck. The blocks in this structure can be considered as fundamental units, with each layer being composed of multiple blocks. By leveraging CNNs, ResNet50 has the capability to extract features at different levels, including low-, mid- and high-level features. It should be noted that increasing the number of layers in the network enables more comprehensive extraction of diverse feature representations. Moreover, as the network depth increasing, it facilitates the extraction of more abstract the features and enhances the semantic information. However, with the original network, simply adding its depth results in gradient dispersion or gradient explosion. Hence, determining the appropriate number of ResNet layers depends on specific requirements.

ResNet34 is a relatively simple network whose basic idea is to build a deep network by adding "residual blocks". By adding cross-layer connections to the network so that information can be passed directly from the previous layer to the subsequent layer. This cross-layer connection can effectively alleviate the problems of gradient disappearance and gradient explosion, which makes the training of the network easier. ResNet34 consists of 34 convolutional layers, including 18 residual blocks. The specific network structure is as follows:

The input layer of ResNet34 comprises a standard convolutional layer with 64 convolutional filters, each having a size of 7 × 7, a stride of 2, and padding of 3. This layer aims to downsample the input image by half while extracting essential low-level features.

In ResNet34, there are 18 residual blocks. Each residual block consists of two convolution layers and a cross-layer connection. Among them, the convolution kernel size of the first convolution layer is 3 × 3, a stride of 1, and padding of 3. The second convolution layer also has a convolution kernel size of 3 × 3, a stride of 1, and a padding of 1. The effect of cross-layer joins is to add the output of the previous layer directly to the input of the later layer. In this way, the information from the previous layer can be retained and passed on to the later layer.

Following the final residual block of ResNet34, a global averaging pooling layer is appended to compute the average output of the last residual block, thereby extracting a comprehensive global feature. This feature encompasses information pertaining to the entire image and can be effectively employed for classification tasks. After the global averaging pooling layer, a fully connected layer is added. The purpose of this layer is to map global features to class scores.

In addition, ResNet34 has a lot of advantages. (1) High precision. The ResNet34 model achieved an impressive classification accuracy of 74.6% on the challenging ImageNet dataset, showcasing its exceptional performance. This remarkable achievement can be attributed to the ingenious design of ResNet, incorporating cross-layer connections and residual blocks, which not only simplifies network training but also significantly enhances its accuracy. (2) the adjusted depth. Residuals and cross-layer connections in ResNet34 enable effective management of network depth. The incorporation of residual blocks allows for increased network depth without encountering issues such as gradient vanishing or exploding. (3) The strong reusability. The utilization of residuals and cross-layer connections in ResNet34 can be extended to other network architectures, thereby enabling the reusability of the ResNet34 model structure for diverse computer vision tasks.

### 3.2 Convolutional block attention module (CBAM)

The attention mechanism (AM), initially applied to machine translation, has now become a pivotal element in neural networks. AM has emerged as an essential component of neural network architecture within the realm of artificial intelligence and finds extensive applications in natural language processing [47], recommendation systems [48], speech recognition [49], computer vision [50], and other fields [51]. AM dynamically focuses on relevant segments of the input that contribute to the current task's execution and combines these concepts of relevance.

The AM module of the convolutional layer, known as CBAM, encompasses the channel AM and spatial AM [52]. Incorporating CBAM into classic networks such as ResNet and MobileNet enhances interpretability and facilitates target recognition. For the input feature map $F\in R^{C\times H\times W}$, *C, H* and *W* represent the number of channels, height and width of the input images respectively, A one-dimensional channel attention feature map $M_{c}\in R^{C\times 1\times 1}$ and two-dimensional spatial attention feature map $M_{s}\in R^{1\times H\times W}$ can be obtained through CBAM, the process is summarized in Eq. (1).

(1) $$\begin{array}{l} F^{\prime }=M_{c}\left(F\right)⊗F\\ F^{\prime \prime }=M_{s}\left(F^{\prime }\right)⊗F^{\prime } \end{array}$$

where ⊗ is element-wise multiplication.

**Fig. 3:**
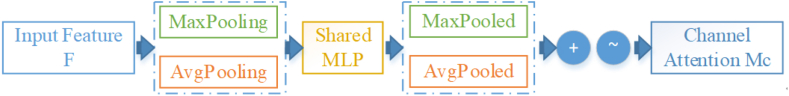
Channel attention module. The weight of each channel of the input image is calculated, and greater attention is allocated to the channel containing crucial information, thereby enhancing the capacity for feature representation.

The channel AM module, depicted in Fig. 3, calculates the weight of each channel in the input image and emphasizes channels containing key information to achieve the feature representation capabilities. To aggregate spatial information from the feature map, both max-pooling and average-pooling operations are employed, generating two different spatial context descriptors: $F_{\max}^{c}$ and $F_{avg}^{c}$ that are average-pooled features and max-pooled features, respectively [53]. In addition, a shared multilayer perceptron receives both descriptors to produce our channel attention map. Subsequently, element-wise summation is used to combine the resultant feature vectors. The hidden activation size is $R^{C/r\times 1\times 1}$, where *r* denotes the reduction ratio, and *C* represents the number of feature channels. The equation for this process is presented in Eq. (2).

(2) $$M_{c}\left(F\right)=\sigma \left(MLP\left(AvgPool\left(F\right)\right)+MLP\left(MaxPool\left(F\right)\right)\right)=\sigma \left(W_{1}\left(W_{0}\left(F_{avg}^{c}\right)\right)+W_{1}\left(W_{0}\left(F_{\max}^{c}\right)\right)\right)$$

where σ is the sigmoid function, $W_{0}\in R^{C/r\times C}$, and $W_{1}\in R^{C\times C/r}$.

**Fig. 4:**
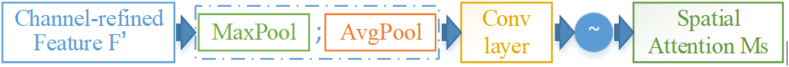
Spatial attention module. The feature descriptor was obtained by applying average pooling and maximum pooling operations along the channel axis, followed by convolution. Finally, the spatial attention feature map was obtained by activating the function.

The spatial AM is illustrated in Fig. 4, which is generated by leveraging the inter-spatial relationship of features. In order to create an effective feature descriptor, we first apply average-pooling and max-pooling procedures along the channel axis and concatenated [54]. Subsequently, a convolutional layer is employed on the concatenated feature descriptor to generate a spatial attention map $M_{s}\left(F\right)\in R^{H\times W}$. The detailed operation process is as follows: two pooling operations are used to aggregate the channel information of a feature map, resulting in generation of two two-dimensional feature maps: $F_{\max}^{s}\in R^{1\times H\times W}$ and $F_{avg}^{s}\in R^{1\times H\times W}$. The features are subjected to max-pooling and average-pooling operationd across the channel, followed by concatenation and convolution with a standard convolutional layer, obtaining a 2D spatial attention feature map. In summary, spatial attention is calculated with Eq. (3).

(3) $$M_{s}\left(F\right)=\sigma \left(f^{7\times 7}\left(\left[AvgPool\left(F\right);MaxPool\left(F\right)\right]\right)\right)=\sigma \left(f^{7\times 7}\left(\left[F_{avg}^{s};F_{\max}^{s}\right]\right)\right)$$

where $f^{7\times 7}$ is a convolution operation with a filter size of 7 × 7.

### 3.3 ResNet34-CBAM

**Fig. 5:**
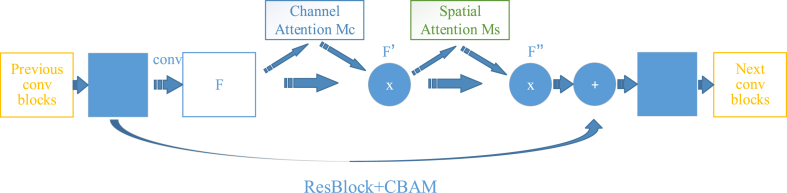
CBAM Convolutional Block Attention Module integrated with ResBlock.

The experiment involved the integration of CBAM into ResNet34. Fig. 5 illustrates the schematic representation of CBAM integrated with a ResBlock, highlighting the accurate positioning of the module within a ResBlock. CBAM was applied to the convolution outputs in each block, and we incorporated CBAM between every ResBlock.

**Fig. 6:**
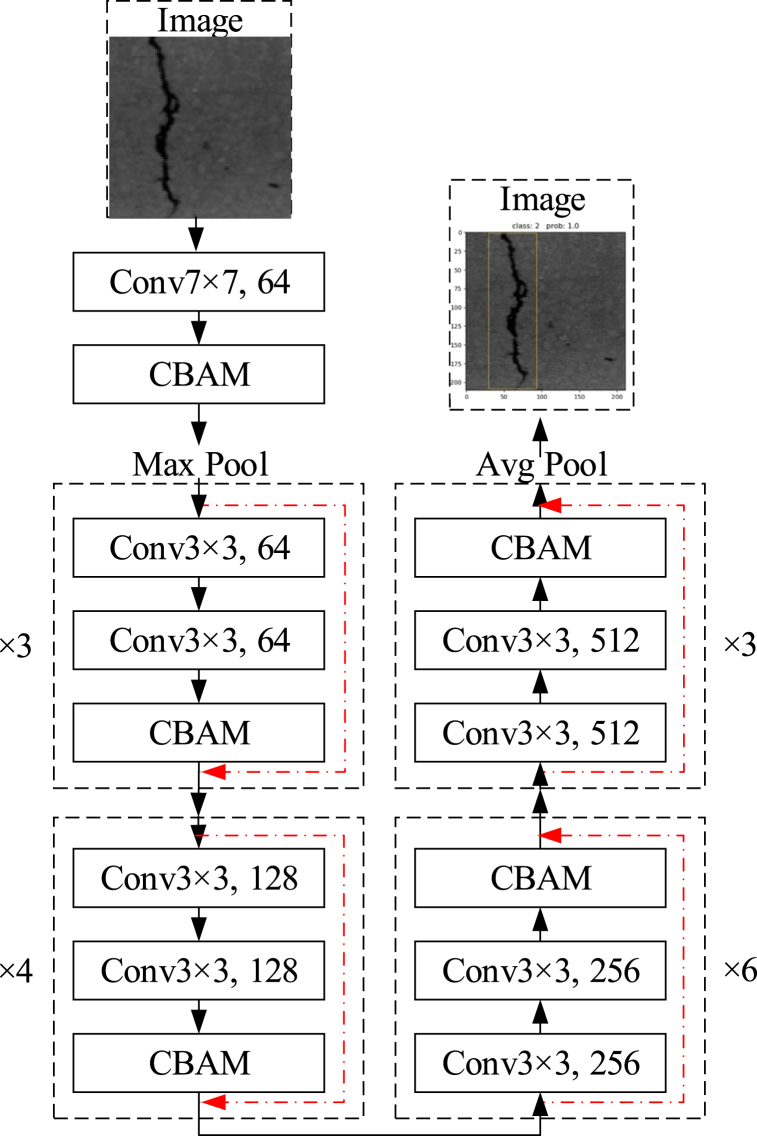
ResNet34 with attention mechanism.

The structure of the ResNet34 network, incorporating the AM proposed in this paper, is illustrated in Fig. 6. The dashed boxes show the residual blocks with different numbers of channel convolutional layers. Each residual block is followed by a channel AM and a spatial AM, denoted as CBAM in the diagram. The right-hand side of each dashed box indicates the number of stacks for residual blocks contained within.

## 4 Datasets

Crack Dataset: The dataset consisted of photographs captured during a comprehensive highway pavement study conducted along a national and provincial highway in Gansu Province, China. Most of the pavement conditions were documented using on-board CCD cameras, which served as the primary data source for this investigation. The pavement images had resolutions of 1688 × 1874. However, due to their large size, these high-resolution images posed challenges in terms of memory requirements for training networks, consequently impeding the learning process. In addition, due to the limited extent of the fracture region within the entire image, extracting distinctive features and accuratedly identify cracks posed a significant challenge.

**Fig. 7:**
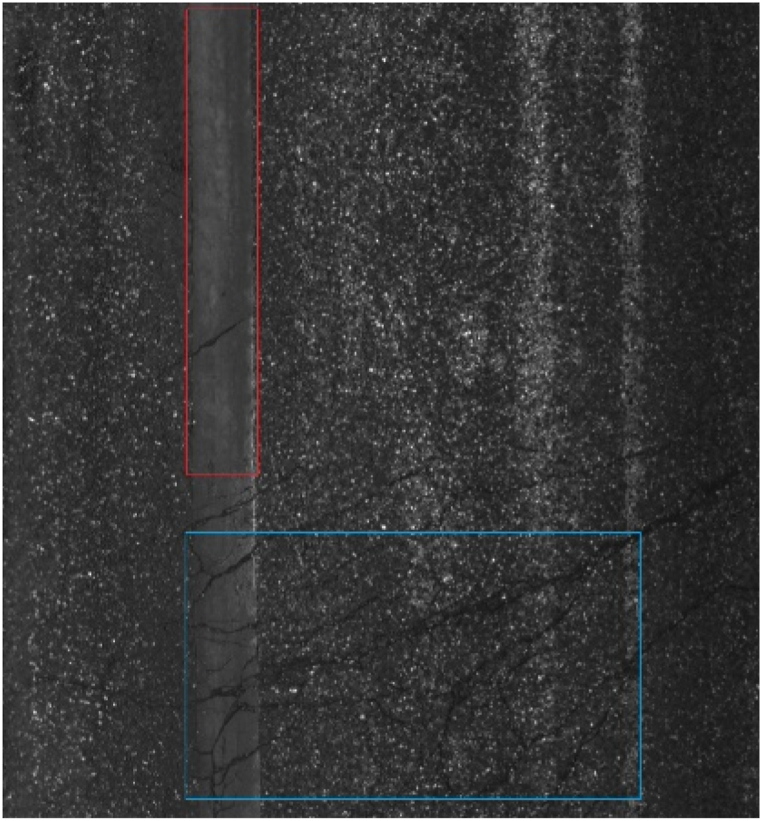
Original pavement images; there are pavement marking and map crack in the red and blue box, respectively.

**Fig. 8:**
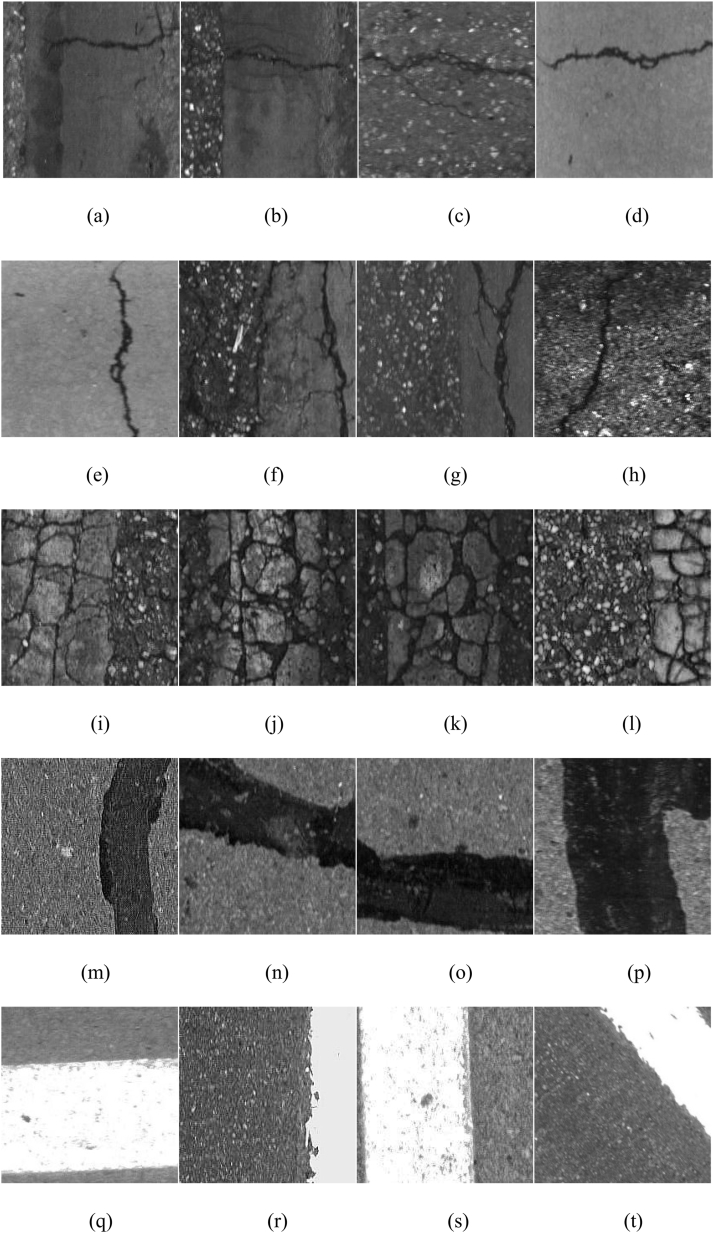
The images of pavement cracks; (a)–(d) Transverse crack; (e)–(h) Longitudinal crack; (i)–(l) Map crack; (m)–(p) Crack repair surface; (q)–(t) Pavement markings.

The original images contained multiple objects (refer to Fig. 7), with pavement marking and map cracks located in the red and blue boxes respectively. To optimize memory usage, minimize interference between classes, and upgrade the precision, we divided the original highway crack images into small blocks measuring 211 × 211 pixels each (see Fig. 8): (a) - (d) represent transverse crack images; (e)-(h) depict longitudinal crack images; (i)-(l) are map crack images; (m) - (p) showcase crack repair surface images; and (q)-(t) exhibit pavement markings images. The dataset was subsequently categorized into six distinct classes manually, namely pavement backgrounds, transverse cracks, longitudinal cracks, map cracks, repairing, and pavement marking. Furthermore, the dataset was partitioned into three subsets for training purposes: a training set, a validation set, and a test set. (see Table 2).

**Table 2:** The dataset.

Class	Training set	Test set	Validation set
pavement backgrounds	10449	3000	1930
transverse cracks	9901	3000	2000
longitudinal cracks	12094	3000	2000
map cracks	8074	3000	2000
repairing	11782	3000	1950
pavement marking	10959	3000	2000

## 5 Experiment

The data underwent several transformations to enhance the model's robustness, including rotation, flip, mirror, and sharp. However, rotation were not applied to transverse and longitudinal cracks. Each experiment was performed employing an NVIDIA GTX 2080Ti GPU with 16 GB of memory. The models were trained and tested using identical hyperparameters: 15 training epochs, an initial learning rate of 0.01, and a weight_decay of 0.0005.

### 5.1 Precision evaluation index

The performance evaluation of the model involved the calculation of precision (P), recall (R), and average Recall (AR) for each category in the test dataset. The formula used to calculate recall is defined as Eq. (4) [49].

(4) $$R=\frac{TP}{TP+FN}$$

where true positive (TP) denotes the correct predicting result and false negative (FN) means the result is not predicted correctly.

### 5.2 Experimental comparison

**Fig. 9:**
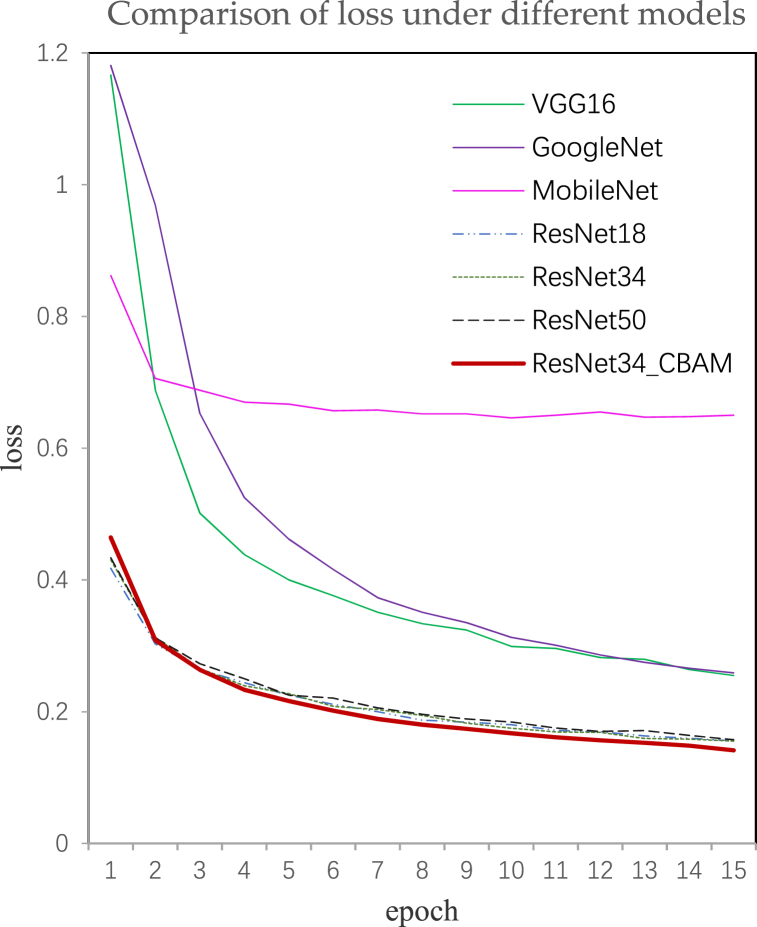
Loss curves under different models' loss. The smaller the value of the function, the lower the loss of the model, the higher the accuracy and the faster the convergence.

Firstly, we trained the ResNet18, ResNet34, and ResNet50 models on the training set. Transfer learning was employed to expedite convergence and upgrade accuracy. Therefore, pre-training models from the ImageNet dataset were utilized for training purposes. The learning rate and epoch were set at 0.0001 and 15, respectively. Subsequently, test precision and average recall were computed for the test set, as presented in Table 3. Notably, both the test precision and the average recall of the ResNet34 model were higher than those of the ResNet18 and ResNet50 models. Therefore, we selected the ResNet34 network as a basic model, and incorporated CBAM into it, resulting in a modified version named ResNet34_CBAM. Subsequently, we evaluated the performance of VGG16, GoogleNet, MobileNet, ResNet18, ResNet34, ResNet50 models along with the proposed ResNet34_CBAM models on the test set by analyzing loss curves, test precision, recall of each class, as well as average recall (refer to Fig. 9 and Table 3, Table 4).

**Table 3:** The test precision and average recall of models.

Model	VGG16	Google Net	Mobile Net	Res Net18	Res Net34	Res Net50	ResNet 34_SE	ResNet 34_CA	ResNet34 _CBAM
P(%)	90.1	91.2	90.6	91.9	92.5	92.1	91.7	92.4	**92.9**
AR(%)	59.8	88.5	68.7	91.5	92.0	90.7	91.9	92.2	**92.5**

**Table 4:** The Recall of each category.

Class Model		transverse	longitudinal	map	repairing	marking
R (%)	VGG16	20.7	19.9	78.5	85.4	94.5
	GoogleNet	82.1	75.4	89.0	96.8	99.2
	MobileNet	53.4	39.4	69.8	87.8	93.2
	ResNet18	82.3	85.7	**92.9**	98.4	98.2
	ResNet34	**90.6**	**87.6**	88.8	93.9	99.0
	ResNet50	87.5	79.1	88.7	**99.1**	99.0
	ResNet34_SE	84.9	64.7	87.6	**99.5**	97.3
	ResNet34_CA	86.3	79.3	88.1	98.4	99.2
	ResNet34_CBAM	88.8	86.8	88.5	98.3	**99.9**

The loss curves under different models are presented in Fig. 9. A smaller value of the function indicates a lower loss of the model and the higher accuracy, resulting in faster convergence. The utilization of pre-training models contributed to the rapid convergence speed observed for each model. Notably, the ResNet34_CBAM model exhibited the lowest loss, suggesting its potential for achieving superior accuracy.

The performance of our model surpassed all other models, as demonstrated in Table 3, achieving the maximum test precision and average recall. Specifically, our proposed model outperformed VGG16, GoogleNet, MobileNet, ResNet18, ResNet34, ResNet50, ResNet34_SE and ResNet34_CA model by 2.8%, 1.7%, 2.3%, 1%, 0.4%, 0.8%, 1.2% and 0.5% respectively in terms of precision. The AR performance of our model was 32.7%, 4%, 23.8%, 1%, 0.5%, 1.8%, 0.6% and 0.3% higher than the VGG16, GoogleNet, MobileNet, ResNet18, ResNet34, ResNet50, ResNet34_SE and ResNet34_CA models respectively. The low AR performance of the VGG16 model can be attributed to its lack of residual structure which leads to overfitting.

The pavement marking crack demonstrated superior performance compared to other types of cracks, as indicated in Table 4. This can be attributed to its larger size and more regular shapes in the images, making it easier to identify. However, there was a slight decrease in the recall of the crack map due to the presence of multiple cracks and unclear features, which could potentially affect precision. The recall rate for transverse cracks in the proposed model was 68.1%, 6.7%, 35.4%, 6.5%, 1.3%, 3.9% and 2.5% higher than that of the VGG16, GoogleNet, MobileNet, ResNet18, ResNet50, ResNet34_SE and ResNet34_CA models models respectively, however, it was found to be lower by only 1.8% compared to the recall rate of the ResNet34 model on transverse cracks. The proposed model exhibited recall rates of 66.9%, 11.4%, 47.4%, 1.1%, 7.7%, 22.1% and 7.5% higher than those achieved by the VGG16, GoogleNet, MobileNet, ResNet18, ResNet50, ResNet34_SE and ResNet34_CA models respectively for longitudinal cracks; however, it demonstrated a slightly lower recall rate (0.8%) compared to that of the ResNet34 model.

In terms of map crack, the proposed model exhibiteda recall rate that was 0.5%, 4.4%, 0.3%, and 0.2% lower than that of the GoogleNet, ResNet18, ResNet34, and ResNet50 models, respectively; however, it demonstrated a higher recall rate by 10%, 18.7%, 0.9% and 0.4% compared to VGG16 MobileNet, ResNet34_SE and ResNet34_CA models respectively. The recall of our model for the crack repair surface was 12.9%, 1.5%, 10.5% and 4.4% higher than that of the VGG16, GoogleNet, MobileNet, and ResNet34 models respectively; however, it was lower by 0.1%, 0.8%, 1.2% and 0.1% compared to the ResNet18, ResNet50, ResNet34_SE and ResNet34_CA model respectively. Our model achieved the highest recall, surpassing VGG16, GoogleNet, MobileNet, ResNet18, ResNet34, ResNet50, ResNet34_SE and ResNet34_CA models by 5.4%, 0.7%, 6.7%, 1.7%, 0.9%, 0.9%, 2.6% and 0.7% respectively in the pavement marking detection task. Furthermore, integrating CBAM into ResNet34, the recall performance for each crack category demonstrates promising results, and the utilization of CBAM facilitates enhanced recognition and extraction of crucial image features by the model, thereby augmenting its overall accuracy.

### 5.3 Recognition results

**Fig. 10:**
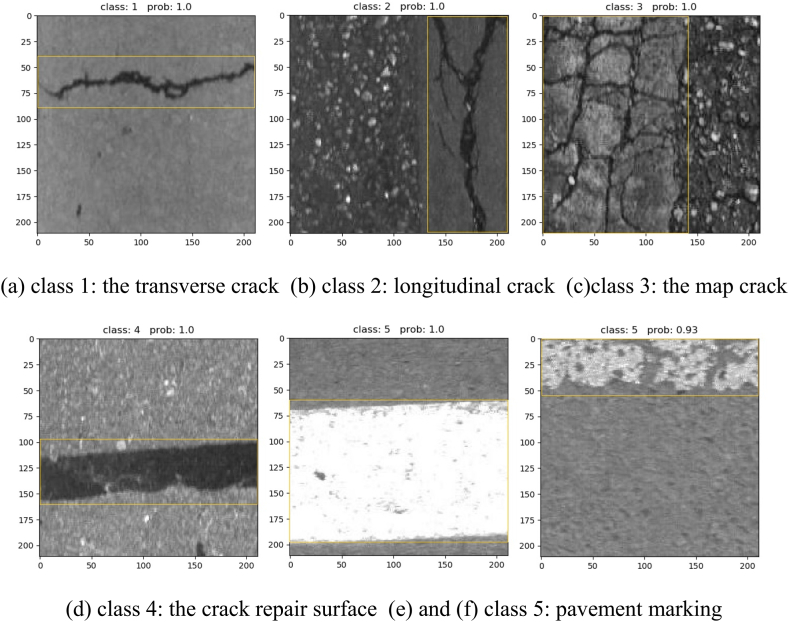
Recognition results.

The proposed model was employed for crack recognition in Fig. 10, where class 1 represents the transverse crack, class 2 denotes the longitudinal crack, class 3 indicates the map crack, class 4 signifies the crack repair surface, and class 5 is the pavement marking.

The adjoint probability, known as Prob., is commonly used in t-tests to indicate the probability that the observed value is exceeded. Prob serves three functions. firstly, it functions as a data test tool encompassing test such as “T test”, “variance analysis”, “co-integration test”, “Granger causality test”, etc. Secondly, it facilitates simple variable processing through calculations of variables such as the “correlation coefficient”, “covariance” and “histogram”. The third aspect involves the application of data simulation, such as the GARCH model cluster. In Fig. 10 (a) indicates a 100% probability of the model correctly identifying the map as a transverse crack; Fig. 10(b) indicates a 100% probability of identifying it as a longitudinal crack; the mesh cracks, repairing, and marking are identified as Fig. 10(c)–(d), and Fig. 10(e) respectively, with a probability of 100%; and Fig. 10(f) represents a 93% probability of identifying it as a pavement marking.

**Fig. 11:**
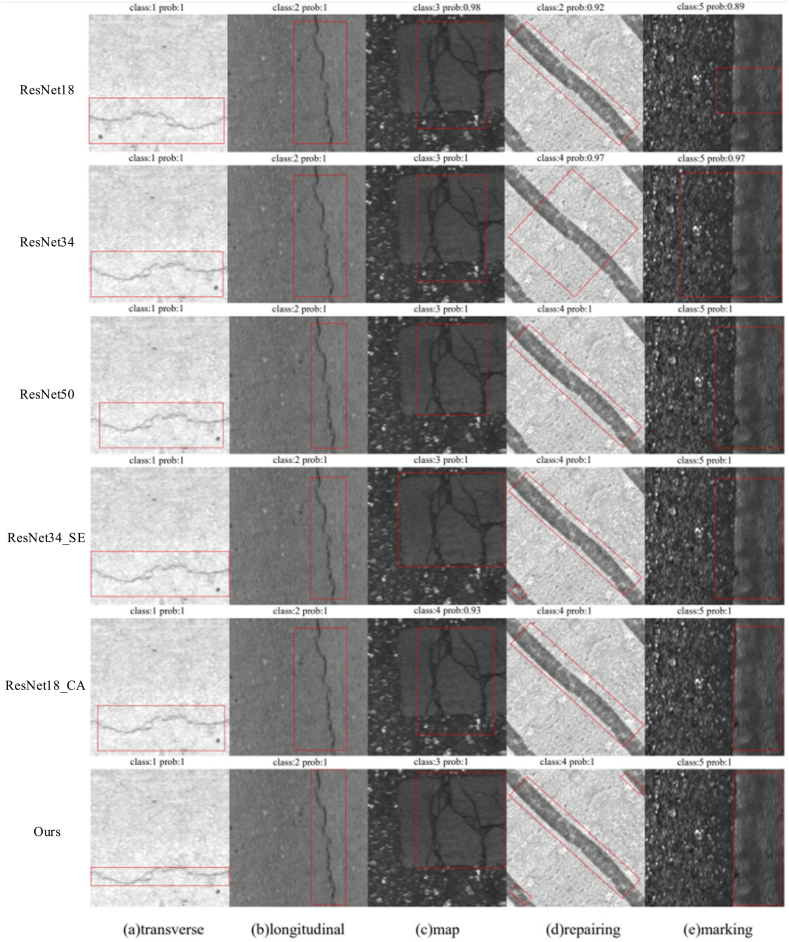
Predicted results of different methods.

Our proposed method demonstrates a significant enhancement in recognition accuracy compared to the other control methods employed, as illustrated in Fig. 11. Notably, both RseNet18 and RseNet34_SE exhibit misclassifications, identifying repairing as longitudinal and mapping as repairing, respectively. Our proposed method shows superior performance in identifying the five different crack categories, each of which can be accurately identified with a prob of 1. Additionally, our proposed method exhibits the highest level of accuracy in calibrating these cracks. Moreover, when it comes to repair identification, only our proposed method accurately detects and identifies the three repairs depicted in the figure.

## 6 Discussion

This paper identifies and extracts various types of crack damage in complex situations, which to some extent aids road maintenance personnel in timely detection of pavement cracks and implementing appropriate repair measures. However, there are certain limitations. Firstly, the detection depth is restricted as the pavement crack detection technology cannot effectively identify underlying diseases beneath the pavement surface. Secondly, the detection of pavement cracks is influenced by weather conditions, atmospheric pollution, and other environmental factors, which can lead to misjudgment or omission of judgment. In our future research, we will focus on identifying pavement diseases in complex climate environments. Additionally, it is crucial to mitigate interference from crack repair surfaces and pavement markings in order to enhance the accuracy of crack recognition.

## 7 Conclusions

The present study applies a novel approach for crack recognition in complex surroundings. Our methodology involves training models through transfer learning and subsequently integrating CBAM into the ResNet34 model. The ensuing discoveries are as follows.

(1) Via adopting the proposed model, the identification and removal of repairing and pavement marking can be implemented to enhance the precision of crack recognition.
(2) Incorporating CBAM into ResBlock not only enhances the test accuracy and average recall of identification, but also improves upon the performance compared with original ResNet34 model.
(3) The precisions of models and recall of each class are consistently high, indicateing that the trained model with ResNet43_CBAM possesses significant utility for recognizing highway cracks in complex environments.

Although this paper identified and extracted crack diseases in complex situations (many different types), the deficiency was that the complex climate environment was not considered. In the future, the identification of pavement diseases in complex climate environments will be taken as our main research content. Meanwhile, it is crucial to suppress interference of the crack repair surface and pavement marking and improve the accuracy of crack recognition.

## Funding

This study was funded by National Key R&D Program of China (2022YFB3903604). The Central Government to Guide Local Scientific and Technological Development(22ZY1QA005). the 10.13039/501100001809National Natural Science Foundation of China (42161069) and was partially supported by 10.13039/501100009014LZJTU EP 201806, Key R&D Project of Gansu Province(21YF11GA008), Science and Technology Program of Gansu Province (23JRRA870) and project of Gansu Provincial Department of Transportation(2021–31). The authors are grateful to the editors and reviewers for their valuable suggestions.

## Ethics approval

Not applicable.

## Consent to participate

Not applicable.

## Consent for publication

Not applicable.

## Code availability

Codes can be obtained from https://github.com/WZMIAOMIAO/deep-learning-for-image-processing and https://github.com/luuuyi/CBAM.PyTorch. And the crack dataset that support the findings of this study are available from the corresponding author upon reasonable request.

## Data availability statement

All data, generated or used during the study are available from the website link. https://drive.google.com/file/d/1j_EQOVj21fKfS4J5iXxWUJE4KjDfakH9/view?usp = drive_link.

## CRediT authorship contribution statement

**Zhihua Zhang:** Writing – review & editing, Writing – original draft, Software, Resources, Methodology, Funding acquisition, Conceptualization. **Kun Yan:** Methodology, Formal analysis, Conceptualization. **Xinxiu Zhang:** Validation, Data curation. **Xing Rong:** Writing – review & editing, Data curation. **Dongdong Feng:** Visualization. **Shuwen Yang:** Investigation.

## Declaration of competing interest

The authors declare that they have no known competing financial interests or personal relationships that could have appeared to influence the work reported in this paper.
